# Uncovering the Predictors of Nonadherence to Insulin Therapy Among Patients With Type 1 and Type 2 Diabetes Attending an Endocrinology Outpatient Clinic of a Tertiary Care Hospital

**DOI:** 10.1016/j.aed.2025.12.011

**Published:** 2025-12-22

**Authors:** Sourav Debnath, Sanjeet Purohit, Anurag Kumar Singh, Pusparghya Pal, Sumit Rajotiya, Shivang Mishra, Prashant Nakash, Sachin Kumar, Shubhadeep Paul, Anupama Sharma, Mahaveer Singh, Deepak Nathiya, Balvir Singh Tomar

**Affiliations:** 1 Department of Pharmacy Practice, Nims Institute of Pharmacy, Nims University Rajasthan, Jaipur, India; 2 Department of Clinical Research, National Institute of Medical Sciences and Research, Nims University Rajasthan, Jaipur, India; 3 Department of Endocrinology, National Institute of Medical Sciences and Research, Nims University Rajasthan, Jaipur, India; 4 Department of Biochemistry, National Institute of Medical Sciences and Research, Nims University Rajasthan, Jaipur, India; 5 Institute of Pediatric Gastroenterology and Hepatology, National Institute of Medical Sciences and Research, Nims University Rajasthan, Jaipur, India

**Keywords:** diabetes mellitus, insulin, medication adherence, polypharmacy, socioeconomic status

## Abstract

**Objective:**

Nonadherence to insulin therapy remains a major challenge to achieving optimal glycemic control, leading to complications and increased health care costs. Despite accessible insulin therapy in tertiary hospitals, barriers such as injection fear, complex regimens, inadequate storage, stigma, and limited health literacy contribute to poor adherence. Evidence on determinants of insulin nonadherence in tertiary care settings in India remains limited. This study aimed to determine the prevalence and predictors of insulin nonadherence among adults with diabetes mellitus.

**Methods:**

A cross-sectional survey was conducted from February to July 2025 among adults (≥18 years) with type 1 or type 2 diabetes mellitus receiving insulin therapy for at least 6 months, selected using a consecutive sampling technique. The 8-item Morisky Medication Adherence Scale assessed adherence; scores <6 indicated nonadherence. Data were analyzed using SPSS version 29, and binary logistic regression identified independent predictors.

**Results:**

Of 402 participants, 36.8% were nonadherent. Independent predictors of nonadherence included upper-middle socioeconomic status (adjusted odds ratio [AOR] 2.11; 95% CI 1.07–4.15; *P* = 0.031), type 2 diabetes (AOR 2.23; 95% CI 1.20–4.14; *P* = 0.011), lack of insulin storage (AOR 4.72; 95% CI 2.28–9.76; *P* < 0.001), polypharmacy (AOR 4.00; 95% CI 0.99–16.14; *P* = 0.046), and poor lifestyle adherence (AOR 1.62; 95% CI 0.95–2.77; *P* = 0.047). Primary education was associated with lower odds of nonadherence (AOR 0.26; 95% CI 0.08–0.83; *P* = 0.023).

**Conclusion:**

Over one-third of patients were nonadherent to insulin therapy. Findings highlight the need for focused educational support, behavioral counseling, and structural solutions such as improved storage provisions to enhance adherence and metabolic outcomes.

**Highlights**

• Over one-third (36.8%) of patients were nonadherent to insulin therapy
• Key independent predictors included type 2 diabetes, upper-middle socioeconomic status, polypharmacy, lack of insulin storage, and poor lifestyle adherence
• Primary education significantly improved adherence, highlighting the importance of health literacy in diabetes management
• Structural and behavioral barriers were more influential than economic constraints in determining adherence
• Targeted education, simplified regimens, and better infrastructural support are essential to improve insulin adherence and glycemic outcomes in tertiary care settings

**Clinical Relevance**

Improving insulin storage access, enhancing patient education, and simplifying treatment regimens can address key adherence barriers identified in this study. These strategies may strengthen insulin use, improve glycemic control, and reduce long-term diabetes complications in Indian tertiary care populations.

## Introduction

Diabetes mellitus is a chronic metabolic disorder characterized by persistent hyperglycemia resulting from impaired insulin secretion, impaired insulin action, or both. It contributes substantially to global morbidity, mortality, and health care expenditure.^1^^,^^2^ According to the International Diabetes Federation, an estimated 537 million adults were living with diabetes worldwide in 2021, and this figure is projected to rise to 783 million by 2045.^3^^,^^4^ India bears the second largest burden globally with more than 101 million adults currently affected and a rapidly rising prevalence.^5^

Type 1 diabetes typically develops during childhood or adolescence due to autoimmune destruction of pancreatic beta cells.^5^ Type 2 diabetes accounts for more than 90% of cases and is primarily associated with modifiable lifestyle factors and population aging.^6^ Although oral antidiabetic medications are frequently used in early type 2 diabetes, insulin therapy is essential for type 1 diabetes and is often required in advanced type 2 diabetes to achieve optimal glycemic control and prevent complications.^7^

Despite its critical importance, adherence to insulin therapy is frequently inadequate, with global estimates suggesting that only around 55% of patients maintain consistent adherence and even lower rates reported in low-resource settings.^8^^,^^9^ Poor adherence contributes to inadequate glycemic control, increased risks of microvascular and macrovascular complications, more frequent hospitalization, and substantial out-of-pocket financial burden in India.^10^, ^11^, ^12^ Barriers to adherence are multifactorial including fear of injections, hypoglycemia concerns, regimen complexity, limited health literacy, inadequate glucometer and storage access, and sociocultural stigma.^13^

Although insulin-specific adherence has been explored in limited Indian studies,^14^ evidence from tertiary care outpatient settings remains scarce. To the best of our knowledge, this is the first from Rajasthan to evaluate insulin adherence in a large cohort including both type 1 and type 2 diabetes patients, using validated assessment tools. The use of a comprehensive analytical approach further strengthens the methodological rigor and distinguishes this work from previous studies. These insights address a critical evidence gap and provide context-specific information to guide targeted interventions.

Objective: To assess the prevalence and predictors of insulin nonadherence among patients with type 1 and type 2 diabetes attending an endocrinology outpatient clinic of a tertiary care hospital in India. Hypothesis: Socioeconomic, behavioral, and structural variables significantly influence insulin nonadherence among adults with diabetes.

## Materials and Methods

### Study Design, Setting, and Period

This hospital-based cross-sectional study was conducted at the Endocrinology Outpatient Department (OPD) of a tertiary care teaching hospital in northern India. Data collection was carried out over a 6-month period, from February 1 to July 31, 2025.

The study was approved by the Institutional Ethics Committee of the participating university. Written informed consent was obtained from all participants prior to enrollment. Confidentiality and anonymity of participant data were strictly maintained, and all procedures complied with the ethical principles outlined in the Declaration of Helsinki (2013 revision).

### Source and Study Population

The source population included all adult patients diagnosed with type 1 or type 2 diabetes mellitus who were on insulin therapy and attended the endocrinology clinic of the study hospital. The study population comprised consecutively recruited patients from this source population who met the inclusion criteria and visited the OPD during the study period.

### Eligibility Criteria

Inclusion criteria were adult patients (aged ≥18 years) with a confirmed diagnosis of type 1 or type 2 diabetes mellitus, who had been on insulin therapy for at least 6 months, and were able to read and understand the local language or could participate with the support of a caregiver.

Exclusion criteria included pregnant or lactating women, individuals with cognitive or psychiatric impairments that limited their ability to participate, patients with active malignancies and severe comorbidities, and those enrolled in other ongoing clinical trials.

#### Sample Size

The sample size was determined using Cochran’s formula for estimating proportions, assuming a 95% confidence level (Z = 1.96), an anticipated prevalence (P) of nonadherence of 43.5% based on a previous study,^15^ and a margin of error (e) of 5%. The formula used was n = Z^2^⋅P⋅(1−P)/e^2^.

Substituting the values, the calculated sample size was 377. To account for an anticipated nonresponse rate of 6%, the final sample size was adjusted to 402 participants. A consecutive sampling technique was employed to recruit eligible patients attending routine follow-up visits at the endocrinology OPD.

#### Missing Data Handling

All questionnaires were reviewed for completeness at the time of data collection. No missing data were identified for any variables included in the analysis. Consequently, all 402 participants had complete responses and were included in the final dataset. No imputation or deletion procedures were required.

### Study Variables

Dependent Variable: Adherence to insulin therapy (adherent vs nonadherent).

Independent Variables: Sociodemographic characteristics, lifestyle and behavioral factors, clinical characteristics, treatment-related factors, and access to health services.

Data Collection Tools and Procedure.

Data were collected through a structured, interviewer-administered questionnaire designed to comprehensively assess factors influencing insulin adherence. The questionnaire comprised 5 sections.

*The first section* captured sociodemographic characteristics, including age (in years), gender (male/female), area of residence (rural/urban), marital status (married/unmarried), socioeconomic status (classified using the Modified Kuppuswamy Scale), education level (illiterate, primary, high school, graduate), occupation (unemployed/employed), salary income (under ₹15 000; ₹15 000–30,000; more than ₹30 000), insurance status (yes/no), and means of conveyance (transport/walking).

*The second section* assessed lifestyle and behavioral factors such as smoking habits, alcohol use (yes/no), adherence to dietary advice (yes/no), regularity of follow-up (yes/no), and the practice of self-monitoring of blood glucose (yes/no).

*The third section* covered clinical characteristics, body mass index categorized according to Asia-specific criteria: underweight (<18.5 kg/m^2^), normal (18.5–22.9 kg/m^2^), overweight (23.0–24.9 kg/m^2^), and obese (≥25.0 kg/m^2^); family history of diabetes (yes/no); presence of comorbidities (yes/no); type of diabetes (type 1/type 2); duration of diabetes mellitus (more than 5 years/less than 5 years); glycated hemoglobin (HbA1c) levels (≥7% or <7%); and frequency of glucose monitoring (occasional/regular).

*The fourth section* explored treatment-related factors, including the number of anti-diabetic medications (≤2 or >2); current treatment regimen (insulin alone or insulin with oral hypoglycemic agents); insulin regimen (bolus, premixed, or basal-bolus); duration of insulin use (less than or more than 5 years); frequency of self-injection (once, twice, or thrice daily); and knowledge about insulin (good/poor). Insulin-related side effects were recorded as hypoglycemia, lipohypertrophy, both, or none.

Lastly, *the fifth section* evaluated access to health services, including the availability of a glucometer at home (yes/no), and any difficulties in insulin administration such as lack of storage facilities, visual impairment, nonavailability of insulin from nearby centers, and polypharmacy.

### Operational Definition

Medication adherence: Medication adherence was assessed using the 8-item Morisky Medication Adherence Scale (MMAS 8), a validated and widely used self-report tool that measures both intentional and unintentional barriers to medication-taking behavior. The scale consists of 8 items with a total score ranging from 0 to 8, where higher scores indicate better adherence. In accordance with standard scoring, participants with scores below 6 were categorized as nonadherent and those with scores of 6 or above were considered adherent. This approach enabled standardized assessment of adherence patterns within the study population.^16^^,^^17^ The questionnaire was pretested among 30 patients attending the endocrinology outpatient clinic to assess clarity, acceptability, and feasibility. Necessary modifications were made based on participant feedback and expert review. The 8-item Morisky Medication Adherence Scale (MMAS-8) demonstrated good internal consistency in this study, with a Cronbach’s α of 0.82, which aligns with previously reported reliability estimates.

Socioeconomic status: Socioeconomic status (SES) was assessed using the Modified Kuppuswamy Scale, a validated tool for determining SES in urban and semiurban populations. The score was calculated based on the education and occupation of the head of household, along with the total monthly family income. The scale assigns weighted scores across these 3 domains, and the combined score categorizes individuals into 5 socioeconomic classes: upper class, upper middle class, lower middle class, upper lower class, and lower class. Income thresholds were updated according to the All-India Consumer Price Index applicable at the time of the study to maintain accuracy. This standardized method enabled an appropriate assessment of the socioeconomic position of participants, which is an important determinant of health behavior and access to care.^18^^,^^19^

### Statistical Analysis

Continuous variables were summarized as mean ± SD, while categorical variables were reported as frequencies and percentages. The normality of continuous data was assessed using the evaluation of skewness and kurtosis. Based on data distribution, independent t-tests were applied for normally distributed (parametric) data and Mann-Whitney U tests for non-normally distributed (nonparametric) data to compare continuous variables. Categorical variables were analyzed using the chi-squared (ꭓ^2^) test.

Variables with a *P* value <0.25 in univariate analysis were considered for inclusion in the multivariable model. Binary logistic regression was used to identify independent predictors of insulin nonadherence. Multicollinearity was assessed using the variance inflation factor, with variables exceeding variance inflation factor >10 excluded. Model selection was guided by the Akaike information criterion to achieve optimal model fit. Adjusted odds ratios (AORs) with 95% CIs were calculated to determine the strength of associations. A two-tailed *P* value <0.05 was considered statistically significant. All analyses were performed using IBM SPSS Statistics version 29 and Microsoft Excel version 1808.

## Result

### Baseline Clinicodemographic Profile

Table 1 summarizes the clinicodemographic and behavioral characteristics of the 402 participants, among whom 148 (36.8%) were nonadherent to insulin therapy (Fig). The overall mean age was 44.9 ± 16.3 years, and nonadherent individuals were significantly younger than adherent ones. Males and rural residents constituted the majority, although nonadherence was relatively more frequent among rural participants.

**Table 1:** Clinic-Demographic Characteristics of the Study Population (*N* = 402)

Variables	Total (*n* = 402)	Adherent (*n* = 254, 63.18%)	Nonadherent (*n* = 148, 36.82%)	*P* value
Age (in years)	44.96 ± 16.27	46.28 ± 16.95	42.70 ± 14.82	**0.033**
Gender				
Male	261 (64.9)	160 (63.0)	101 (68.2)	0.287
Female	141 (35.1)	94 (37.0)	47 (31.8)	
Area of residence				
Rural	341 (84.8)	223 (87.8)	118 (79.7)	**0.030**
Urban	61 (15.2)	31 (12.2)	30 (20.3)	
Marital status				
Married	276 (68.7)	175 (68.9)	101 (68.2)	
Unmarried	126 (31.3)	79 (31.1)	47 (31.8)	0.891
Socioeconomic status				
Lower class	133 (33.1)	95 (37.4)	38 (25.7)	**<0.001**
Upper lower class	116 (28.9)	76 (29.9)	40 (27.0)	
Lower middle class	79 (19.7)	51 (20.1)	28 (18.9)	
Upper middle	74 (18.4)	32 (12.6)	42 (28.4)	
Education status				
Illiterate	89 (22.1)	59 (23.2)	30 (20.3)	**0.002**
Primary	42 (10.4)	37 (14.6)	5 (3.4)	
High school	245 (60.9)	143 (56.3)	102 (68.9)	
Graduate	26 (6.5)	15 (5.9)	11 (7.4)	
Occupation				
Unemployed	183 (45.5)	124 (48.8)	59 (39.9)	0.082
Employed	219 (54.5)	130 (51.2)	89 (60.1)	
Insurance status				
Yes	85 (21.1)	55 (21.7)	30 (20.3)	0.743
No	317 (78.9)	199 (78.3)	118 (79.7)	
Salary income				
Under 15 000	247 (61.4)	165 (65.0)	82 (55.4)	0.068
Between 15 000 to 30 000	124 (30.8)	68 (26.8)	56 (37.8)	
More than 30 000	31 (7.7)	21 (8.3)	10 (6.8)	
Means of conveyance				
Transport	393 (97.8)	246 (96.9)	147 (99.3)	
Walking	9 (2.2)	8 (3.1)	1 (0.7)	0.106
BMI category				
Underweight	34 (8.5)	20 (7.9)	14 (9.5)	0.870
Normal	181 (45.0)	115 (45.3)	66 (44.6)	
Overweight	80 (19.9)	53 (20.9)	27 (18.2)	
Obese	107 (26.6)	66 (16.4)	41 (27.7)	
Smoking habits				
Yes	111 (27.6)	66 (26.0)	45 (30.4)	0.339
No	291 (72.4)	188 (74.0)	103 (69.6)	
Alcohol habits				
Yes	93 (23.1)	54 (21.3)	39 (26.4)	0.243
No	309 (76.9)	200 (78.7)	109 (73.6)	
Family history of diabetes				
Yes	139 (34.6)	83 (32.7)	56 (37.8)	0.294
No	263 (65.4)	171 (67.3)	92 (62.2)	
Any comorbidities				
Yes	176 (43.8)	115 (45.3)	61 (41.2)	0.429
No	226 (56.2)	139 (54.7)	87 (58.8)	
Frequency of glucose monitoring				
Occasional	192 (47.8)	111 (43.7)	81 (54.7)	**0.033**
Regular	210 (52.2)	143 (56.3)	67 (45.3)	
Number of antidiabetics				
More than 2	55 (13.7)	35 (13.8)	20 (13.5)	0.940
Less than 2	347 (86.3)	219 (86.2)	128 (86.5)	
Knowledge about insulin				
Poor	183 (45.5)	109 (42.9)	74 (50.0)	0.169
Good	219 (54.5)	145 (57.1)	74 (50.0)	
Side effects of insulin administration				
Hypoglycemia	37 (9.2)	27 (10.6)	10 (6.8)	**0.010**
Lipohypertrophy	36 (9.0)	22 (8.7)	14 (9.5)	
Both (Hypoglycemia with Lipohypertrophy)	24 (6.0)	22 (8.7)	2 (1.4)	
None	305 (75.9)	183 (72.0)	122 (82.4)	
Types of diabetes				
Type 1	133 (33.1)	107 (42.1)	26 (17.6)	**<0.001**
Type 2	269 (66.9)	147 (57.9)	122 (82.4)	
Duration of DM				
More than 5 y	165 (41.0)	121 (47.6)	44 (29.7)	**<0.001**
Less than 5 y	237 (59.0)	133 (52.4)	104 (70.3)	
Current treatment regimen				
Insulin	364 (90.5)	225 (88.6)	139 (93.9)	0.078
Insulin + OHA	38 (9.5)	29 (11.4)	9 (6.1)	
Insulin regimen				
Bolus	259 (64.4)	164 (64.6)	95 (64.2)	0.550
Premixed	103 (25.6)	62 (24.4)	41 (27.7)	
Basal bolus	40 (10.0)	28 (11.0)	12 (8.1)	
Duration of insulin intake				
Less than 5 y	302 (75.1)	195 (76.8)	107 (72.3)	0.317
More than 5 y	100 (24.9)	59 (23.2)	41 (27.7)	
Having a glucometer at home				
Yes	221 (55.0)	155 (61.0)	66 (44.6)	**<0.001**
No	181 (45.0)	99 (39.0)	82 (55.4)	
Frequency of self-injection				
Once a day	12 (3.0)	8 (2.0)	4 (1.0)	0.599
Twice a day	100 (24.9)	59 (14.7)	41 (10.2)	
Thrice a day	290 (72.1)	103 (25.6)	103 (25.6)	
Any difficulties in taking insulin				
Lack of facility for storage	50 (12.4)	16 (6.3)	34 (23.0)	**<0.001**
Visual impairment	20 (5.0)	15 (5.9)	5 (3.4)	
Nonavailability of insulin from nearby	17 (4.2)	11 (4.3)	6 (4.1)	
Polypharmacy	19 (4.7)	13 (5.1)	6 (4.1)	
No difficulty	296 (73.6)	199 (78.3)	97 (65.5)	
Practice SMGB				
Yes	197 (49.0)	132 (52.0)	65 (43.9)	0.119
No	205 (51.0)	122 (48.0)	83 (56.1)	
Regular follow-up				
Yes	247 (61.4)	160 (63.0)	87 (58.8)	0.403
No	155 (38.6)	94 (37.0)	61 (41.2)	
Dietary advice followed				
Yes	308 (76.6)	205 (80.7)	103 (69.6)	**0.011**
No	94 (23.4)	49 (19.3)	45 (30.4)	
HbA1c level (%)				
More than or equal to 7	388 (96.6)	245 (96.5)	143 (96.6)	0.931
Less than 7	14 (3.5)	9 (5.5)	5 (3.4)	

Abbreviations: DM = diabetes mellitus; HbA1c, glycated hemoglobin; OHA = oral hypoglycemic agent; BMI = body mass index; SMGB = self-monitoring blood glucose.

Bold values indicate statistically significant results (*P* value <0.05 was considered statistically significant). Continuous variables were compared using the independent t test or Mann–Whitney U test depending on normality. Categorical variables were analyzed using the chi-square test.

©MMAS 2006. All Rights Reserved. MMAS-8 content protected by U.S. copyright laws. Permission granted by Donald E. Morisky, ScD, MSPH, ScM.

**Fig:**
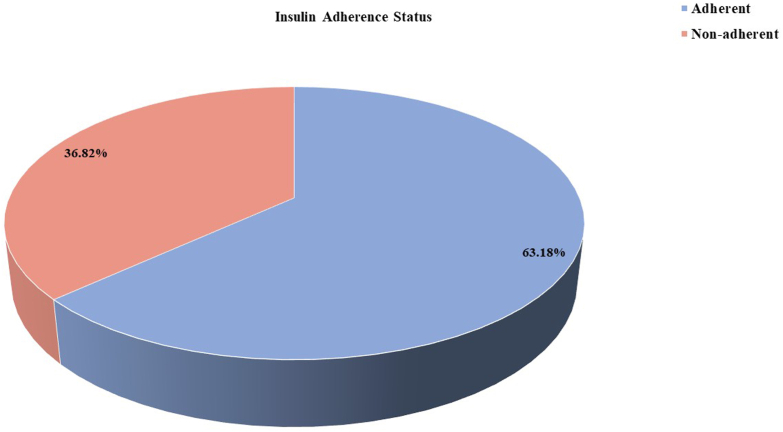
Level of adherence to insulin therapy among the study participants.

Socioeconomic and educational gradients were evident: participants from the upper-middle socioeconomic class and those with lower educational attainment demonstrated higher nonadherence rates. Type 2 diabetes and a shorter disease duration (<5 years) were significantly associated with nonadherence.

Behavioral practices also differed between groups. Adherent individuals more often self-monitored blood glucose and possessed a home glucometer. Conversely, nonadherent participants more frequently reported barriers such as lack of insulin storage facilities, drug unavailability, and polypharmacy. Adherence was also higher among those following dietary and lifestyle recommendations. Although most participants (96.6%) had suboptimal glycemic control (HbA1c ≥ 7%), no significant difference in HbA1c levels was observed between groups.

#### Predictors Associated With Nonadherence to Insulin Therapy

In univariate logistic regression analysis, sixteen variables were associated with nonadherence (*P* < 0.25) (Table 2). After adjusting for confounders, 6 variables remained independent predictors in the multivariate model (Table 3).

**Table 2:** Univariate Logistic Regression Analysis for Predictors Associated With Nonadherence to Insulin Therapy Among Study Participants

Variables	COR (95% CI)	*P* value
Age (in years)	0.986 (0.974-0.999)	**0.034**
Gender		
Male	Ref.	
Female	0.792 (0.515-1.217)	0.288
Area of residence		
Rural	Ref.	
Urban	1.829 (1.056-3.168)	**0.031**
Marital status		
Married	Ref.	
Unmarried	0.970 (0.627-1.501)	0.891
Socioeconomic status		
Lower class	Ref.	**0.001**
Upper lower class	1.316 (0.769-2.251)	0.316
Lower middle class	1.373 (0.757-2.489)	0.297
Upper middle	3.281 (1.811-5.944)	0.052
Family history of diabetes		
Yes	Ref.	0.294
No	1.254 (0.821-1.915)	
Any comorbidities		
Yes	Ref.	0.429
No	0.847 (0.562-1.277)	
Education status		
Illiterate	Ref.	**0.006**
Primary	0.266 (0.095-0.746)	**0.012**
Highschool	1.403 (0.844-2.331)	0.191
Graduate	1.442 (0.590-3.525)	0.422
Occupation		
Unemployed	Ref.	0.083
Employed	1.439 (0.954-2.170)	
Insurance		
Yes	Ref.	0.743
No	1.087 (0.660-1.792)	
Means of conveyance		
Transport	Ref.	0.142
Walking	4.780 (0.592-38.606)	
Frequency of glucose monitoring		
Regular	Ref.	**0.033**
Occasional	1.557 (1.036-2.341)	
Number of antidiabetics		
More than 2	Ref.	0.940
Less than 2	1.023 (0.566-1.847)	
Knowledge about insulin		
Poor	Ref.	0.169
Good	0.752 (0.500-1.129)	
Side effects of insulin administration		
Hypoglycemia	Ref.	**0.029**
Lipohypertrophy	1.718 (0.640-4.613)	0.283
Both (Hypoglycemia with Lipohypertrophy)	0.245 (0.049-1.239)	0.089
No	1.800 (0.841-3.852)	0.130
Type of diabetes		
Type 1	Ref.	**<0.001**
Type 2	3.415 (2.090-5.582)	
Duration of DM		
More than 5 y	Ref.	**<0.001**
Less than 5 y	2.150 (1.399-3.306)	
Treatment regimen (type-2 only)		
Insulin + OHA	Ref.	0.083
Insulin	0.502 (0.231-1.093)	
Insulin regimen		
Basal	Ref.	0.552
Premixed	1.142 (0.715-1.824)	0.580
Basal bolus	0.740 (0.359-1.523)	0.413
Duration of insulin intake		
Less than 5 y	Ref.	0.317
More than 5 y	1.266 (0.797-2.012)	
Having a glucometer at home		
Yes	Ref.	**0.001**
No	1.945 (1.290-2.933)	
Frequency of self-injection		
Once a day	Ref.	0.600
Twice a day	1.390 (0.392-4.923)	0.610
Thrice a day	1.102 (0.324-3.747)	0.877
Any difficulties in taking insulin		
No difficulty	Ref.	0.436
Lack of facility for storage	4.360 (2.294-8.283)	**<0.001**
Visual impairment	0.684 (0.242-1.936)	0.474
Non-availability of insulin from nearby	1.119 (0.402-3.115)	0.830
Polypharmacy	0.947 (0.349-2.567)	0.915
Practice SMGB		
No	Ref.	0.120
Yes	1.382 (0.919-2.076)	
Regular follow-up		
No	Ref.	0.403
Yes	1.193 (0.788-1.807)	
Adherence to diet and exercise		
Yes	Ref.	**0.012**
No	1.828 (1.144-2.921)	
HbA1c (%)		
More than or equal to 7	Ref.	
Less than 7	1.051 (0.313-2.895)	0.931

Abbreviations: COR = crude odds ratio; DM = diabetes mellitus; HbA1c, glycated hemoglobin; OHA = oral hypoglycemic agent; SMGB = self-monitoring blood glucose.

Bold values indicate statistically significant results (*P* value <0.05 was considered statistically significant). Binary logistic regression was used to compute crude odds ratios (COR) with 95% CI. Variables with *P* value <0.25 in univariate analysis were considered for multivariable modeling.

*Reference category*: adherence group.

**Table 3:** Multivariate Logistic Regression Analysis for Predictors Associated With Nonadherence to Insulin Therapy

Variables	AOR (95% CI)	*P* value
Age (in years)	0.995 (0.979-1.011)	0.541
Area of residence		
Rural	Ref.	0.297
Urban	0.717 (0.384-1.339)	
Socioeconomic status		
Lower class	Ref.	0.167
Upper lower class	1.169 (0.626-2.183)	0.167
Lower middle class	1.389 (0.693-2.787)	0.177
Upper middle	2.110 (1.073-4.149)	**0.031**
Education status		
Illiterate	Ref.	**0.023**
Primary	0.259 (0.081-0.827)	0.078
Highschool	1.105 (0.609-2.002)	0.743
Graduate	1.060 (0.389-2.887)	0.909
Occupation		
Unemployed	Ref.	0.262
Employed	1.328 (0.809-2.179)	
Means of conveyance		
Transport	Ref.	0.532
Walking	2.073 (0.211-20.367)	
Frequency of glucose monitoring		
Regular	Ref.	0.156
Occasional	1.420 (0.874-2.305)	
Side effects of insulin administration		
Hypoglycemia	Ref.	0.176
Lipohypertrophy	2.272 (0.694-7.435)	0.175
Both (Hypoglycemia with Lipohypertrophy)	0.427 (0.073-2.496)	0.345
None	1.793 (0.709-4.537)	0.217
Type of diabetes		
Type 1	Ref.	**0.011**
Type 2	2.233 (1.204-4.139)	
Duration of DM		
More than 5 y	Ref.	0.285
Less than 5 y	1.332 (0.788-2.253)	
Treatment regimen		
Insulin + OHA	Ref.	0.233
Insulin	0.539 (0.196-1.486)	
Having a glucometer at home		
Yes	Ref.	0.242
No	1.353 (0.815-2.245)	
Any difficulties in taking insulin		
No difficulty	Ref.	**<0.001**
Lack of facility for storage	4.721 (2.284-9.757)	**<0.001**
Visual impairment	0.848 (0.253-2.842)	0.790
Non-availability of insulin from nearby	1.702 (0.498-5.822)	0.396
Polypharmacy	3.996 (0.989-16.139)	**0.046**
Practice SMGB		
Yes	Ref.	0.408
No	0.808 (0.487-1.340)	
Adherence to diet and exercise		
Yes	Ref.	**0.047**
No	1.621 (0.948-2.772)	

Abbreviations: AOR = adjusted odds ratio; DM = diabetes mellitus; OHA = oral hypoglycemic agent; SMGB = self-monitoring blood glucose.

Bold values indicate statistically significant results (*P* value <0.05 was considered statistically significant). Binary logistic regression was used to compute adjusted odds ratios (AOR) with 95% CI. Multicollinearity was assessed using the variance inflation factor.

*Reference category:* adherence group.

Participants with type 2 diabetes had more than twice the odds of being nonadherent compared with those with type 1 diabetes (AOR = 2.23; 95% CI: 1.20–4.14; *P* = 0.011). Belonging to the upper-middle socioeconomic class also increased the likelihood of nonadherence (AOR = 2.11; 95% CI: 1.07–4.15; *P* = 0.031). In contrast, having primary education reduced the odds of nonadherence by 74% compared with being illiterate (AOR = 0.26; 95% CI: 0.08–0.83; *P* = 0.023).

Structural and treatment-related barriers played a crucial role: lack of insulin storage was associated with nearly fivefold higher odds of nonadherence (AOR = 4.72; 95% CI: 2.28–9.76; *P* < 0.001), while polypharmacy increased the odds approximately fourfold (AOR = 4.00; 95% CI: 0.99–16.14; *P* = 0.046). Additionally, not adhering to dietary and exercise recommendations was independently associated with a higher likelihood of nonadherence (AOR = 1.62; 95% CI: 0.95–2.77; *P* = 0.047).

## Discussion

The current study provides valuable insights into the prevalence and determinants of insulin nonadherence among individuals with type 1 and type 2 diabetes in an outpatient tertiary care center in India. The observed nonadherence rate of 36.8% aligns with global figures, which indicate that approximately 45% to 55% of patients do not consistently adhere to their insulin therapy regimens.^9^ Beyond quantifying nonadherence, this study contributes to a deeper understanding by interpreting how demographic, socioeconomic, behavioral, and structural determinants interact to influence insulin-taking behavior.

A key finding is the substantially higher odds of insulin nonadherence among individuals with type 2 diabetes compared to those with type 1 diabetes. This disparity may reflect differing perceptions of the disease; patients with type 2 diabetes often do not view insulin as an essential part of their treatment, particularly in the early stages. Prior studies have shown that reluctance to initiate or sustain insulin therapy in this group is influenced by psychological insulin resistance, social stigma, and the misconceptions that insulin use reflects worsening disease severity or personal inadequacy.^8^^,^^10^ These factors may collectively contribute to reduced motivation and greater hesitancy toward insulin adherence in type 2 diabetes.

Interestingly, the study found an unexpected association between higher socioeconomic status, particularly among the upper-middle class, and insulin nonadherence. While it is typically assumed that individuals from lower-income backgrounds are more vulnerable due to limited access to medications, our findings suggest that affluent individuals may face distinct barriers. These include rigid work schedules, denial of illness, lifestyle constraints, and a lower responsiveness to public health messaging. This observation aligns with findings from the National Family Health Survey, which highlights that greater awareness and access to treatment do not necessarily lead to better diabetes control across all socioeconomic groups in India. Similarly, Fernandez-Lazaro et al reported that medication nonadherence is prevalent across the socioeconomic spectrum, affecting both low-income, uninsured patients and those with more resources.^3^^,^^20^ Comparable South Asian evidence by Wahiduzzaman et al^21^ demonstrates that sociodemographic characteristics significantly influence medication adherence among individuals with diabetes, reinforcing that adherence challenges are not confined to economically disadvantaged groups.

Educational attainment emerged as a protective factor against insulin nonadherence, with participants who had at least a primary education being more likely to adhere to their insulin regimen than those who were illiterate. This underscores the critical role of health literacy in managing chronic conditions. Even basic education enhances patients' understanding of insulin therapy, its proper usage, self-monitoring, and the importance of lifestyle changes.^14^ Afni similarly reported that health literacy is a key determinant of treatment adherence and self-care behaviors in chronic conditions.^22^ These observations are consistent with the behavior change communication framework described by Darukaradhya and Krishnamurthy,^23^ who emphasize that culturally tailored and patient-centered communication strategies are vital for improving adherence to long-term therapies, including insulin.

Behavioral and structural factors also play a crucial role in insulin adherence. The study revealed a significant association between nonadherence and the lack of proper insulin storage facilities. Participants without adequate storage were over 4 times more likely to be nonadherent. This highlights a major infrastructural challenge, particularly in resource-constrained settings where reliable refrigeration is not available. Since insulin requires cold-chain storage to remain effective, this barrier disproportionately affects rural and low-income households, further exacerbating existing health disparities.^12^

Additionally, polypharmacy was identified as an independent predictor of insulin nonadherence. Patients managing multiple medications were nearly 4 times more likely to skip insulin doses. Cognitive overload, forgetfulness, and the perceived burden of taking numerous medications often lead patients to prioritize those that provide immediate symptom relief. This finding highlights the need for integrated care approaches, simplified treatment regimens, and the use of supportive tools, such as pillboxes, reminder systems, and pharmacist-led interventions, particularly for patients with multiple health conditions.^13^

Adherence to lifestyle measures, including diet and physical activity, was also associated with better insulin adherence. This reinforces the understanding that medication adherence is part of a broader commitment to managing one's health. Comprehensive diabetes education programs that address diet, physical activity, glucose monitoring, and insulin use as interconnected components are critical to improving overall diabetes management.^24^

Interestingly, urban residence was initially associated with higher nonadherence in univariate analysis, though this association lost significance in the multivariate model. While urban areas are typically thought to offer better access to health care, they may introduce challenges such as time constraints, occupational stress, and limited family support, all of which can disrupt routine self-care behaviors. This trend suggests that further qualitative research is needed to better understand the specific barriers to adherence in urban settings.

Furthermore, poor glycemic control was evident across the cohort, with 96.6% of participants exhibiting HbA1c levels ≥7%, regardless of their adherence status. The absence of significant difference in HbA1c between adherent and nonadherent groups may be explained by the long-term nature of HbA1c, which reflects average glycemia over the preceding two to 3 months. In contrast, self-reported adherence captures more recent behavior that may not yet translate into measurable changes in HbA1c. Additionally, recall bias in reporting adherence may further contribute to this observation. These findings suggest that insulin adherence alone may be insufficient to achieve optimal metabolic control, particularly in settings where multiple clinical and behavioral factors influence glycemic outcomes.

Evidence from previous studies supports this perspective. Haghighatpanah et al reported that factors such as female gender, older age, high-density lipoprotein cholesterol levels, duration of diabetes, and type of medication were significant predictors of glycemic control.^25^ Taken together, these insights underscore the need for a more comprehensive diabetes management approach that integrates consistent monitoring, timely dose adjustments, and structured lifestyle interventions to address the multifactorial determinants of glycemic control.

### Strengths of the Study

This study is one of the few to comprehensively assess insulin adherence in both type 1 and type 2 diabetes within an outpatient tertiary care context in India. The study utilized a robust statistical approach, including multivariate logistic regression guided by the Akaike information criterion and variance inflation factor, to optimize model fit and reduce bias due to multicollinearity. The inclusion of validated predictors and detailed sociodemographic profiling enhances the relevance of the findings for similar tertiary care settings in low- and middle-income countries.

Importantly, to the best of our knowledge, this is the first study to simultaneously include both type 1 and type 2 diabetic patients to examine predictors associated with insulin nonadherence. This work contributes to the limited but growing body of research on insulin-specific adherence in India, addressing an important gap in the literature that has traditionally focused on oral antidiabetic agents.^8^ By identifying modifiable barriers such as education level, challenges in insulin storage, and adherence to lifestyle recommendations, the study provides potential targets for future intervention, although further research is required to evaluate the effectiveness of these measures.

## Limitations

Despite its strengths, this study has several limitations. First, the cross-sectional design limits the ability to draw causal inferences; while associations were identified, their directionality cannot be determined. Second, adherence was assessed through self-reported behaviors, which are subject to recall and social desirability bias. Objective measures such as pharmacy refill data, electronic monitoring, or longitudinal HbA1c trends may offer stronger validation of adherence patterns.

Third, the sample was drawn from a tertiary care hospital, so the findings may not be generalizable to rural primary care settings or private health care facilities where patient characteristics and service availability may differ. Fourth, psychological and cultural factors such as diabetes-related distress, stigma surrounding insulin use, and mental health status were not assessed, although these may influence adherence. Finally, qualitative insights into patient experiences were not captured; such data could provide a deeper understanding of contextual barriers.

Future research should aim to include longitudinal study designs, test structured interventions, and incorporate mixed-methods approaches to explore behavioral, cultural, and gender-specific determinants of adherence. Extending this research framework to other low-resource settings could further strengthen global understanding of insulin adherence.

### Public Health and Clinical Implications

This study highlights the need for practical, context-appropriate interventions to improve insulin adherence in tertiary care settings. Strengthening literacy-sensitive diabetes education, expanding telemonitoring and digital reminder systems, and improving access to reliable insulin storage solutions can directly address major barriers identified in this population. Simplifying treatment regimens where clinically feasible and adopting a multidisciplinary care approach involving pharmacists, nurse educators, and counselors may further support sustained adherence. Collectively, these strategies can enhance patient engagement, improve glycemic outcomes, and reduce the long-term burden of diabetes complications.

## Conclusion

With over one-third of participants identified as non-adherent, this study highlights the complex and multifactorial nature of insulin adherence in tertiary care settings. Key predictors such as type 2 diabetes, higher socioeconomic status, lower educational attainment, inadequate insulin storage, polypharmacy, and inconsistent lifestyle practices operate through interconnected behavioral, structural, and contextual pathways that influence patients’ ability to adhere to insulin therapy.

These findings emphasize the need for targeted educational strategies, improved storage support, simplified treatment regimens, and integrated lifestyle counseling to strengthen adherence. Future longitudinal and interventional research is needed to determine the effectiveness of these approaches. At the policy level, strengthening diabetes education programs, expanding telehealth supported monitoring and investing in accessible storage infrastructure may play an important role in improving insulin adherence and reducing the long-term burden of diabetes in India.

## Institutional Review Board Statement

This study was conducted in accordance with the ethical standards of the Declaration of Helsinki and was approved by the Institutional Ethical Committee of the National Institute of Medical Sciences and Research, Rajasthan, Jaipur (Approval No. NIMSUR/IEC/2025/1387).

## Informed Consent Statement

Written informed consent was obtained from all participants prior to their inclusion in the study.

## Data Availability Statement

The data supporting the findings of this study are available from the corresponding author upon reasonable request.

## Disclosure

The authors have no conflicts of interest to disclose.
